# Regulation of Metabolic Plasticity in Cancer Stem Cells and Implications in Cancer Therapy

**DOI:** 10.3390/cancers14235912

**Published:** 2022-11-30

**Authors:** Styliani Papadaki, Angeliki Magklara

**Affiliations:** 1 Department of Clinical Chemistry, Faculty of Medicine, University of Ioannina, 45110 Ioannina, Greece; 2 Biomedical Research Institute–Foundation for Research and Technology, 45110 Ioannina, Greece; 3 Institute of Biosciences, University Research Center of Ioannina (URCI), 45110 Ioannina, Greece

**Keywords:** cancer stem cells, metabolism, metabolic plasticity, metabolic reprogramming, aerobic glycolysis, glycolysis, oxidative phosphorylation, autophagy, hypoxia, drug resistance

## Abstract

**Simple Summary:**

Glucose is the main fuel cell used for energy production via a series of enzymatic reactions in the presence of oxygen in a process known as aerobic respiration. The main steps in this process are glycolysis, the tricarboxylic acid cycle, and oxidative phosphorylation (OXPHOS). Cancer cells rely mostly on glycolysis and less on OXPHOS for rapid production of energy and intermediate macromolecules that are required to sustain their increased proliferation rate. This metabolic reprogramming is considered one of the hallmarks of cancer and has been linked to tumor growth and progression, as well as to the development of therapy resistance. Cancer stem cells (CSCs) are a subset of tumor cells with self-renewal and differentiation capacities and have gained much attention due to their involvement in cancer initiation and resistance to conventional therapies. In contrast to the bulk of tumor cells, CSCs can switch between glycolysis and OXPHOS depending on stimuli from their microenvironment. This metabolic plasticity allows them to adapt and survive under various stressful conditions, maintaining, at the same time, their stemness properties, and, thus, contributing to the development of therapy resistance and tumor recurrence. Consequently, understanding the specific features of CSC metabolism is crucial for the successful elimination of these cells. In this review, we provide a concise description of the metabolic signatures of CSCs, emphasizing their metabolic plasticity and its involvement in drug resistance; we also draw attention to the potential of targeting CSC metabolism as a complementary therapeutic approach in cancer.

**Abstract:**

Cancer stem cells (CSCs), a subpopulation of tumor cells with self-renewal capacity, have been associated with tumor initiation, progression, and therapy resistance. While the bulk of tumor cells mainly use glycolysis for energy production, CSCs have gained attention for their ability to switch between glycolysis and oxidative phosphorylation, depending on their energy needs and stimuli from their microenvironment. This metabolic plasticity is mediated by signaling pathways that are also implicated in the regulation of CSC properties, such as the Wnt/β-catenin, Notch, and Hippo networks. Two other stemness-associated processes, autophagy and hypoxia, seem to play a role in the metabolic switching of CSCs as well. Importantly, accumulating evidence has linked the metabolic plasticity of CSCs to their increased resistance to treatment. In this review, we summarize the metabolic signatures of CSCs and the pathways that regulate them; we especially highlight research data that demonstrate the metabolic adaptability of these cells and their role in stemness and therapy resistance. As the development of drug resistance is a major challenge for successful cancer treatment, the potential of specific elimination of CSCs through targeting their metabolism is of great interest and it is particularly examined.

## 1. Introduction

Cancer is the second leading cause of death globally, accounting for an estimated 9.6 million deaths in 2018 according to the World Health Organization (WHO) [1]. It is a remarkably heterogeneous disease with tumors of the same type showing distinct molecular and histopathological features across patients (inter-tumor heterogeneity) [2] and cell populations within the same tumor having discrete genetic makeup and phenotype (intra-tumor heterogeneity) [3]. There are two prevailing concepts that explain intra-tumoral heterogeneity, the stochastic or clonal evolution model and the cancer stem cell (CSC) model [4].

The clonal evolution model maintains that all tumor cells are initially biologically equivalent. The accumulation of genetic and epigenetic alterations in some tumor cells may result in functionally and phenotypically distinct clones with different degrees of aggressiveness, invasiveness and/or therapy resistance [5]. The second model attributes tumor heterogeneity to a small subpopulation of tumor cells, namely the CSCs that are characterized by self-renewal and the potential to differentiate into multiple lineages [6]. CSCs are believed to be responsible for tumor initiation and progression with the experimental proof provided by their ability to form tumors in immunodeficient mice in very low numbers [7]. Several lines of evidence also suggest that CSCs are resistant to conventional anti-cancer treatments and failure to target them can result to tumor relapse and metastasis [8]. On the other hand, the differentiated cancer cells (non-CSCs) that form the bulk of the tumor have no self-renewal capacity, are non-tumorigenic, and are more susceptible to standard therapeutic schemes than CSCs. Consequently, the research community has focused its efforts on the elucidation of the biologically distinct identity of CSCs for the development of targeted therapies against them. An integral part of the CSC unique identity is their metabolome, which varies considerably from that of their non-CSC counterparts [9].

The term “metabolism” encompasses a large group of intracellular, complex chemical reactions that use nutrients for energy production and macromolecule synthesis and are indispensable for all cellular functions. Healthy and cancer cells share mostly common metabolic pathways [10]; however, certain adaptations are required in the latter to meet their high demands in energy and macromolecules for their increased proliferation and growth rate [10]. Glycolysis and glucose metabolism are the main metabolic pathways that are known to be altered in cancer cells [10]. Indeed, the most prominent characteristic of cancer cell metabolism is considered to be the high dependency on glucose, one of the main fuel molecules for adenosine triphosphate (ATP) production in the cell [11]. 

Notably, intra-tumoral heterogeneity is also manifested on the metabolic level, with subpopulations of tumor cells exhibiting distinct metabolic characteristics [12]. Contradictory data from different studies had presented CSCs either as more glycolytic or as more dependent on oxidative phosphorylation (OXPHOS) for their energy needs [13]. The current understanding is that CSCs possess metabolic flexibility triggered by external stimuli, allowing them to adapt to various conditions so that they can survive and maintain their stemness properties [13]. This metabolic plasticity is considered one of the hallmarks of CSCs that differentiate them from non-CSCs [14]. 

In this review, we aim to provide a concise description of the distinctive metabolic traits of CSCs and the pathways that regulate them, as well as discuss how these are implicated in promoting stemness. We particularly highlight studies that demonstrate the metabolic plasticity of CSCs and its role in drug resistance, and we present research data that underline the promise of targeting CSC metabolism as a complementary therapeutic approach to alleviate the burden of cancer.

## 2. Cancer Stem Cell Metabolism—Glycolysis or Oxidative Phosphorylation?

**Figure 1:**
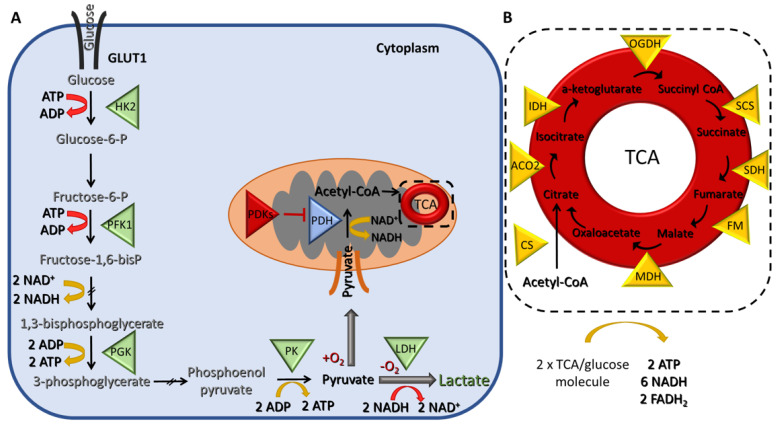
Schematic representation of glycolysis and the tricarboxylic acid (TCA) cycle (**A**) During glycolysis, two molecules of adenosine triphosphate (ATP) are consumed and four are produced, resulting in a gain of two molecules of ATP per one molecule of glucose. In addition, two molecules of nicotinamide adenine dinucleotide (NADH) are also generated. Under hypoxic conditions, pyruvate is further fermented into lactate resulting in the regeneration of two molecules of NAD^+^ that can be used again in glycolysis. (**B**) Under normoxic conditions, pyruvate is converted to acetyl-CoA in the mitochondria, which then enters the TCA cycle, where two turns are needed to process one molecule of glucose leading to the generation of two molecules of ATP, six NADH and two flavin adenine dinucleotide (FADH_2_). Triangles indicate enzymes. Abbreviations: (**A**) GLUT1; glucose transporter 1, HK2; hexokinase 2, PKF1; phosphofructokinase 1, PGK; phosphoglycerate kinase, PK; pyruvate kinase, LDH; lactate dehydrogenase, PDH; pyruvate dehydrogenase, PDKs; pyruvate dehydrogenase kinases. (**B**) CS; citrate synthase, ACO2; aconitase, IDH; isocitrate dehydrogenase, OGDC; oxoglutarate dehydrogenase, SCS; succinyl-CoA synthase, SDH; succinate dehydrogenase, FM; fumarate hydratase, MDH; malate dehydrogenase.

Cells break down glucose to produce ATP for their energy needs through a series of chemical reactions that are collectively known as cellular respiration. The first step is glycolysis, and it takes place in the cytoplasm, where glucose is converted to pyruvate (Figure 1A). 

**Figure 2:**
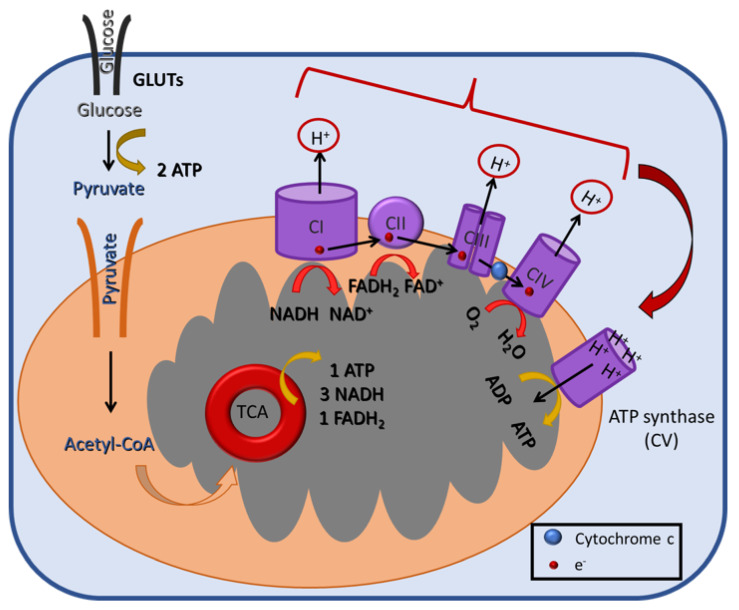
A schematic illustration of the mitochondrial oxidative phosphorylation (OXPHOS) process in the inner mitochondrial membrane. During OXPHOS (aka electron transport-linked phosphorylation), cells oxidize metabolites and release energy in the form of ATP. The electrons produced from the reduction of NADH by Complex I (CI) or of FADH_2_ by CII are transferred through CIII and CIV to the terminal electron acceptor, O_2_. These reactions create a H^+^ gradient across the mitochondrial inner membrane, which is harvested by ATP synthase (or CV) for the generation of ATP.

The net energy production of glycolysis is two molecules of ATP and two of reduced nicotinamide adenine dinucleotide (NADH) per glucose molecule. In the presence of oxygen, the pyruvate is transferred to the mitochondria, where it is converted to acetyl-CoA, which, in turn, enters the tricarboxylic acid (TCA) cycle, a chain of chemical reactions that leads to its oxidation to CO_2_ and the release of three molecules of NADH, one FADH_2_, and one ATP (or GTP) (Figure 1B). The TCA cycle is closely linked with the process of OXPHOS, the final step of aerobic respiration, which also takes place in the inner membrane of the mitochondria. The NADH and FADH_2_ produced during the previous step are now utilized for electron transfer in a series of oxidation–reduction reactions that ultimately lead to the generation of 36 ATPs/glucose molecules (Figure 2). Under hypoxic conditions, the glycolytic pathway is favored, and the pyruvate remains in the cytoplasm, where it is converted to lactate, a process referred to as anaerobic glycolysis (aka pyruvate fermentation) that yields only two ATPs/glucose molecules (Figure 1A). 

**Figure 3:**
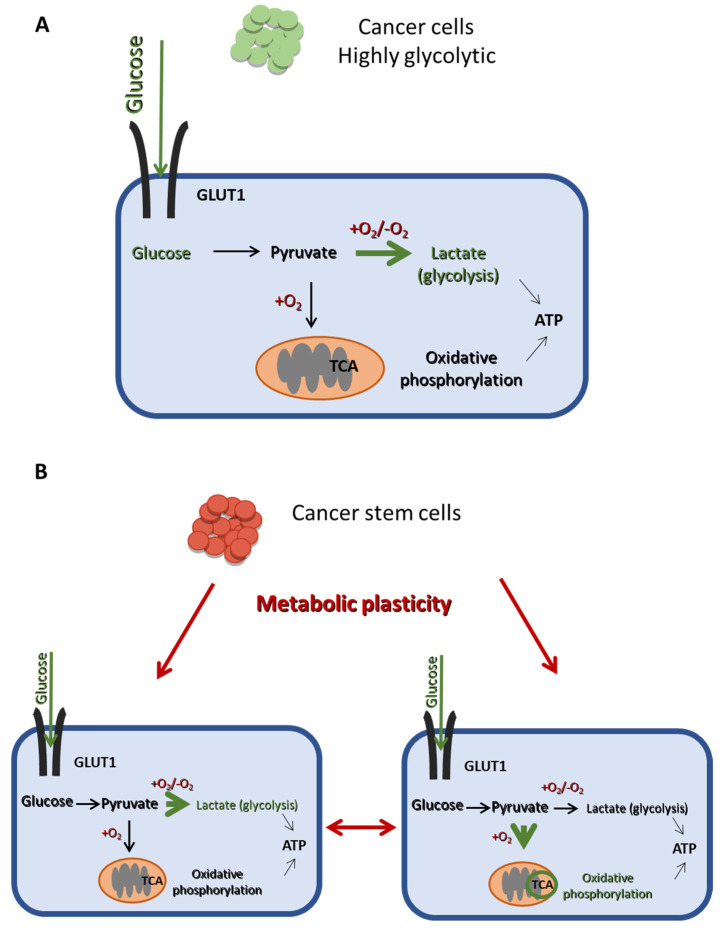
The main metabolic difference between CSCs and non-CSCs. (**A**) Cancer cells are mainly dependent on glycolysis for energy production, even in the presence of oxygen, as indicated by the thick green arrow, while they undergo OXPHOS in a lesser extend (thin black arrow). (**B**) CSCs exhibit a metabolic plasticity and can switch from glycolysis to OXPHOS and vice vera, depending on their energy demands and external stimuli. Thus, CSCs can produce ATP using mainly either glycolysis or OXPHOS, as indicated by the thick green arrows in the right and the left panel, respectively.

Cancer cells prefer to convert glucose to lactate, irrespectively of the presence of oxygen, a phenomenon first described by Warburg and known as aerobic glycolysis [11]. Notably, cancer cells still carry out some OXPHOS, but to a much lesser extent (Figure 3A). A main advantage of aerobic glycolysis is the rapid production of ATP, albeit a less efficient one, since only two ATPs are produced per glucose molecule. To make up for this deficit, glucose uptake by cancer cells is abnormally high and is supported by the upregulation of glucose transporter 1 (GLUT1) [15]. Furthermore, other key enzymes and proteins mediating the Warburg effect, such as monocarboxylate transporter 1 and 4 [16], hexokinase 2 (HK2), lactate dehydrogenase A (LDHA), and pyruvate dehydrogenase kinase 1 (PDK1), have also been reported to be overexpressed in different cancers [14,17].

Whereas the bulk of tumor cells mainly use aerobic glycolysis for glucose metabolism, CSCs can exhibit metabolic flexibility and switch between glycolysis and OXPHOS (Figure 3B), depending on their energy needs and environmental stimuli [14,18]. This metabolic plasticity is a critical difference between CSCs and non-CSCs (Figure 3) and may be partly mediating the stemness and therapy resistance properties of the former. 

### 2.1. Glycolysis in CSCs

Several studies have shown that CSCs derived from various tumor types are even more glycolytic than non-CSCs, as they express higher levels of glycolysis-associated genes and lower levels of genes involved in OXPHOS [19,20,21,22,23,24,25]. 

Indeed, ALDH^+^ breast CSCs purified from MDA-MB-231 and MCF-7 mammospheres expressed higher levels of the glycolytic gene *PDK1* and reduced levels of pyruvate dehydrogenase (*PDH*) compared to non-CSCs [19]. Knock-down of *PDK1* in MDA-MB-231 cells impaired the stemness properties of CSCs, leading to a decline in the ALDH^+^-subpopulation and reducing mammosphere forming efficiency (MFE) and downregulation in the expression of stemness genes [19]. A different research group using the same in vitro system confirmed that switching from OXPHOS to aerobic glycolysis was essential for CSCs to maintain their stemness properties through a decrease in the levels of reactive oxygen species (ROS) [20]. Specifically, overexpression of the rate limiting enzyme of glyconeogenesis fructose-1,6-bisphosphatase (FBP1) led to an increase in ROS levels and suppression of tumorsphere formation and expression of CSC markers. In contrast, loss of FBP1 led to reprogramming from OXPHOS to glycolysis, reduced ROS levels, and enhancement of CSC traits and tumorigenicity [20]. These observations were also confirmed by independent proteomic and targeted metabolomic analyses, which showed that breast CSCs, derived from specimens from patients undergoing surgery, shifted from OXPHOS to anaerobic glycolysis, as they exhibited higher lactic fermentation, higher levels of glycolysis intermediates, and upregulation of the key glycolytic enzymes pyruvate kinase M2 (PKM2) and LDHA compared to non-CSCs [21]. The use of doxorubicin against breast CSCs confirmed their increased resistance to this drug, as it only had a cytostatic effect with the cells being blocked in the G2 phase of the cell cycle. However, when CSCs were treated with the glycolysis inhibitor 2-deoxy-D-glucose (2-DG), alone or in combination with doxorubicin, their viability was significantly reduced [21]. 

In hepatocellular carcinoma (HCC), the CD133^+^ CSCs, isolated from the PLC/PRF/5 cell line, exhibited upregulation of the glycolytic genes *GLUT1*, *HK2*, *PDK4,* and *PGM1* (phosphoglucomutase 1) and downregulation of the gluconeogenic genes *G6Pase* (glucose-6 phospatase) and *PEPCK* (phosphoenolpyruvate carboxykinase), leading to decreased cellular ATP levels compared to CD133^−^ non-CSCs [22]. Glycolysis inhibition resulted in diminished stemness properties and sphere-formation ability in the CD133^+^ CSCs, further supporting the idea that enhanced glycolysis plays a significant role in hepatic CSC maintenance [22].

Similar results were also obtained with cultures of PAMC-82 and SNU16 spheroids enriched in gastric CSCs, which showed high levels of the glycolytic enzyme enolase 1 (ENO1) [23]. Overexpression of *ENO1* enhanced the glycolytic capacity, as well as the stemness properties, of the gastric CSCs. Glycolysis inhibition using 2-DG led to impairment of the self-renewal, migratory, and invasive capacities of these cells, further highlighting the link between stemness and glycolysis. Notably, high ENO1 expression was also associated with poor patient prognosis [23].

In accordance with the above observations, side-population cells with CS-like characteristics isolated from the non-small cell lung cancer (NSCLC) A549 cell line by flow cytometry also bore a hyperglycolytic profile, as indicated by the higher glucose uptake and lactate production, as well as by the higher expression of glycolytic enzymes (including PDK-1 and HK-1) compared to differentiated cancer cells [24]. Furthermore, CSC-enriched tumorspheres from the NSCLC H460 cell line overexpressed manganese superoxide dismutase (MnSOD) [25], a mitochondrial antioxidant enzyme that protects cells from oxidative stress, but has also been shown to promote a metabolic shift to glycolysis in cancer cells [26]. Knockout of MnSOD resulted in suppression of glycolysis and of the stem-like traits in lung CSCs [25]. MnSOD was proposed to upregulate key glycolytic enzymes and to contribute to a metabolic switch from OXPHOS to glycolysis in these cells [25].

Overall, the above-described studies suggest that the high glycolytic activity in CSCs is interlinked with their stemness properties. Thus, targeting glycolysis could be a promising therapeutic approach to eliminate this aggressive cancer sub-population. 

### 2.2. Oxidative Phosphorylation in CSCs

An increased rate of glycolysis is not always the rule in CSCs, since a number of other studies have demonstrated a preference of these cells towards OXPHOS for energy production to sustain their survival [27,28,29,30,31,32,33]. 

A prime example of CSCs that favor OXPHOS to meet their energy demands are the glioma stem cells (GSCs). GSCs isolated from neurospheres generated from the U87, GBM-146, and GBM-176 cell lines appeared less glycolytic than the differentiated glioma cells, as they consumed less glucose and produced more lactate, while they relied more on OXPHOS to yield higher ATP levels [27]. 

Similarly, in CD34^+^ leukemic stem cells (LSCs) derived from patients with chronic myeloid leukemia, metabolic analysis revealed increased levels of OXPHOS compared to CD34^−^ cells, while inhibition of this process resulted in their selective eradication in vitro [28]. In a different study, the signal transducer and activator of transcription 3 (STAT3) was shown to be a mediator of OXPHOS in LSCs derived from primary human acute myeloid leukemia (AML) specimens [29]. STAT3 is known to regulate the expression of *MYC,* which in turn controls the transcription of the amino-acid transporter *SLC1A5* that is also implicated in the regulation of glutaminolysis. Inhibition of any of the above proteins in LSCs led to reduced TCA cycle activity and inhibition of OXPHOS, establishing the STAT3-MYC-SLC1A5 axis as a regulator of energy metabolism in these cells. The authors also showed a potential therapeutic application of their data by using a small molecule STAT3 inhibitor, which led to the selective death of stem and progenitor cells isolated from AML patients, while sparing normal hematopoietic cells [29].

Metabolic heterogeneity has also been observed between CD133^+^/CD44^+^ liver CSCs (LCSCs), derived from the HCCLM3 HCC cell line, and their differentiated counterparts, with the former exhibiting more robust levels of OXPHOS [30]. Indeed, the downregulation of LDHA, the increased levels of pyruvate, and those of the three subunits of the PDH complex (PDHC), as well as the higher mitochondria number, strongly argued that LCSCs preferably used OXPHOS for energy production [30]. This process seemed to be crucial for maintaining their stemness potential. Glycolysis inhibition after treatment of LCSCs with 2-DG resulted in the enhanced expression of stemness genes and of the cell surface markers CD133 and CD44. It also led to upregulation of pyruvate levels and overexpression of *PDHC*, indicating the involvement of OXPHOS in the stemness potential of the cells. Inhibition of OXPHOS by the inhibitor of mitochondrial division Mdivi-1 led to downregulation of stemness genes and of CD133 and CD44, further supporting the role of this process in the maintenance of LCSCs [30]. 

Likewise, in patient-derived ovarian CD44^+^/CD117^+^ CSCs, the overexpression of the OXPHOS genes *PDHK1* and *PDH* and the higher mitochondrial activity suggested a preference for pyruvate fueling the TCA cycle and for OXPHOS over glycolysis [31]. OXPHOS inhibition dramatically affected the survival of CD44^+^/CD117^+^ CSCs without affecting the viability of CD44^+^/CD117^−^ cells [31]. 

Even though the targeting of oncogene-driven signaling pathways represents a clinically validated therapeutic approach, a fraction of surviving cells leads to tumor relapse. Based on this observation, in an interesting study by Viale et al., the authors tried to illuminate the role of *KRAS*, a well-known oncogene in pancreatic ductal adenocarcinoma (PDAC), in cancer maintenance [32]. They showed that only a small portion of dormant tumor cells survived KRAS ablation (called surviving cells, SCs), and these cells had stem-like characteristics and were responsible for tumor relapse. Transcriptomic and metabolomic analyses of the stem-like SCs revealed that they relied more on OXPHOS for their energy needs, while glycolysis was impaired [32]. Inhibition of OXPHOS resulted in a decrease in sphere formation and was effective against tumor recurrence, suggesting that this process was also indispensable for SC survival and maintenance [32]. 

In small cell lung cancer, CSCs were isolated from the H446 cell line by sorting the urokinase-type plasminogen activator receptor (uPAR) positive cells, since this receptor is associated with CSC function [33]. These cells maintained a low state metabolic activity and were less dependent on aerobic glycolysis, as it was indicated by the lower glucose uptake and lactate production compared to non-CSCs [33]. Suppression of OXPHOS had a greater impact in ATP production compared to glycolysis inhibition in the lung CSCs, supporting their preference towards OXPHOS to meet their energy demands [33]. 

In conclusion, even though CSCs lie in hypoxic niches, they may still prefer OXPHOS to glycolysis in some cases. This paradoxical phenomenon may be attributed to two reasons: a) the metabolic symbiosis of non-CSCs with CSCs could result in the use of the lactate produced via glycolysis by the former for the OXPHOS of the latter, leading to an impressively efficient way of fuel utilization; b) contrary to the bulk of tumor cells, CSCs are generally maintained in a quiescent state with low proliferative activity and, therefore, do not need glycolysis intermediates for macromolecule biosynthesis [33]. 

### 2.3. Metabolic Heterogeneity and Plasticity in CSCs

As it was mentioned before, several studies have demonstrated that CSCs have the ability to alter their metabolic phenotype as a response to signals from the stromal niche or to external stressors, such as drug exposure [13].

Metabolic heterogeneity seems to be a feature of pancreatic CSCs (PaCSCs) [34]. In a very thorough study, Sanco et al. showed that the majority of these cells were highly dependent on OXPHOS and displayed low metabolic plasticity, but a subset of PaCSCs that survived and expanded after metformin treatment, was characterized by an intermediate phenotype and marked metabolic adaptability [34]. The authors used PaCSC-enriched tumorspheres derived from primary cultures of patient-derived xenografts to show that they depended more on OXPHOS and less on glycolysis compared to non-CSCs, since RNA-sequencing data revealed an increased expression for several TCA enzymes and mitochondrial OXPHOS components [34]. These data were also confirmed by RT-PCR in sorted CD133^+^ CSCs. Along the same lines, both spheres and CD133^+^ CSCs exhibited increased mitochondrial mass, but lower glucose uptake, lactate production, and ROS levels [34]. Interestingly, metformin inhibition of OXPHOS led to reduced ATP levels and increased apoptosis, suggesting that these PaCSCs had reduced metabolic plasticity, as they were not able to switch to glycolysis to counterbalance the loss of ATP [34]. However, upon prolonged metformin treatment, some PaCSCs survived, and they were characterized by high glucose uptake and lactate production, suggesting high glycolytic activity. These resistant CSCs could also influx glucose into the mitochondria and use OXPHOS for ATP production, showing an intermediate metabolic phenotype [34]. Metformin treatment could not induce apoptosis in these cells, suggestive of increased metabolic plasticity, since the cells could rely on enhanced glycolysis for their survival and had become less susceptible to OXPHOS targeting. Metformin withdrawal reversed the metabolic phenotype of the resistant PaCSCs back to that of the sensitive ones confirming their enhanced metabolic plasticity that allowed them to adapt to environmental changes. The authors went on to show that the molecular mechanism underlying the metabolic switching in the resistant cells was the *MYC* overexpression. *MYC* was downregulated in the drug-sensitive CSCs, and it was moderately expressed in the resistant PaCSCs, where it negatively regulated the peroxisome proliferator-activated receptor-gamma coactivator 1A (PGC1A), which is essential for mitochondrial metabolism [34]. Inhibition of *MYC* expression could restore the resistance to metformin by enforcing PaCSC dependance towards OXPHOS through PCG1A upregulation. Thus, metabolic heterogeneity of PaCSCs should be considered for the design of efficient therapies against them [34].

In another interesting study, Luo et al. isolated two types of breast CSCs from the SUM149, HCC1806, MCF-7, and T47D cell lines that carried two distinct metabolic profiles [35]. The first type was characterized by a proliferative epithelial-like state (E) that highly expressed ALDH (ALDH^+^ CSCs) and exhibited increased OXPHOS; the second type was characterized by a quiescent, invasive mesenchymal-like state (M) that highly expressed CD44 (CD44^+^ CSCs) and was more glycolytic [35]. A hypoxic or oxidative stress could lead to the transition from the M to the E state, an effect that was reversible when an antioxidant was used, indicating that CSCs were not locked in one metabolic state, but they could show metabolic adaptability according to external stimuli [35]. 

Metabolic heterogeneity in CSCs has been an ongoing field of intense research, as the lack of a common pattern makes their metabolic characterization a challenging matter. The above results highlight the fact that elucidation of the metabolic signatures of all CSC subpopulations in a tumor is mandatory for their effective eradication; targeting both OXPHOS and glycolysis may constitute a better therapeutic strategy against them [36]. 

## 3. Other Metabolic Pathways in CSCs

### 3.1. Pentose Phosphate Pathway

Glycolysis is connected with the pentose phosphate pathway (PPP) that uses glycolysis intermediates for macromolecule biosynthesis to support cancer cell proliferation. The catalytic action of HK2 results in the phosphorylation of glucose with the product, glucose-6-P, entering the two phases of PPP in the cytosol. The first phase is oxidative and results in the conversion of glycose-6-P into ribulose-5-phosphate (Ru-5-P) and the production of nicotinamide adenine dinucleotide phosphate (NADPH). NADPH is essential for the maintenance of a redox balance under stress, as it is implicated in ROS elimination. Cancer cells use this as an antioxidant mechanism; in response to high ROS levels, they can enhance glycolysis and promote the PPP to generate more NADPH. The second phase of PPP is non-oxidative and results in the conversion of Ru-5-P either into ribose-5-phosphate, which is essential for nucleic acid synthesis, or into xylulose-5-phosphate that generates the glycolytic intermediates fructose 6-phosphate and glyceraldehyde 3-phosphate, which are precursors of amino acid synthesis [37]. 

CSCs exhibit increased glucose influx into the PPP, which serves to meet their high anabolic demands, to regulate oxidative stress and in the development of chemoresistance. Treatment with 5-fuorouracil (5-FU) or oxaliplatin of *KRAS* mutant colorectal carcinomas in mice led to the enrichment of CUB-domain-containing protein 1 positive (CDCP1^+^) cells [38]. CPCP1 is a type I transmembrane glycoprotein that has been found upregulated in several solid cancers and has been associated with disease progression and poor patient survival [39]. The CDCP1^+^ CSCs showed increased oxidative PPP metabolite levels and de novo purine biosynthesis mediated by the inactivation of the glycolytic enzyme triosephosphate isomerase. The metabolic rerouting towards PPP protected CDCP1^+^ CSCs from the oxidative stress induced by chemotherapy, while targeting the oxidative phase of PPP resulted in increased chemosensitivity of the cells [38].

In an in vitro model of breast cancer, histone deacetylase (HDAC) inhibitors could reprogram non-breast CSCs into stem-like cells by promoting PPP metabolism [40]. Specifically, HDAC-induced CSCs derived from the SUM159 cell line showed high glucose consumption with no change in lactate levels, as well as higher NADPH levels, supporting the notion that these cells possessed an enhanced PPP metabolism compared to non-CSCs [40]. The use of PPP inhibitors significantly reduced MFE both in ER^+^ (MCF7, T47D) and ER^−^ (SUM149, SUM159) cell lines [40], suggesting that this pathway regulated cancer stemness.

The aforementioned studies draw attention to another metabolic route that may be enhanced in CSCs, shedding more light on the metabolic heterogeneity of these tumor cells. 

### 3.2. Glutamine Metabolism

Glutamine (Gln) is a nonessential amino acid, as it is endogenously synthesized, and can either fuel the TCA cycle leading to other amino acid and glutathione (GSH) biosynthesis, or it can remain in the cytosol and be used for nucleotide synthesis and production of glutamate in the process [41]. When the fueling of the TCA cycle with pyruvate is limited due to high lactate production, Gln can enter the TCA cycle, where it is converted to α-ketoglutarate (α-KG), and it can lead both to ATP production and to replenishment of TCA cycle intermediates. During hypoxic conditions, α-KG can be converted to citrate that can exit from mitochondria to the cytosol, where it is used for fatty acid synthesis and NADPH production. Additionally, cytosolic glutamate through GSH production is crucial for redox homeostasis and oxidative stress protection [41]. The effect of Gln metabolism in the regulation of CSCs is yet under investigation; here, we provide some insight into the role of Gln in the stemness properties of CSCs.

A study conducted in prostate cancer cell lines revealed that Gln was significantly upregulated in radioresistant DU145, but not in radioresistant LNCaP cells, compared to the parental ones [42]. Inhibition of Gln metabolism resulted in radiosensitization of the former, while activation of autophagy protected LNCaP cells from the radiation effects under Gln deprivation. DU145 ALDH^+^ CSCs and tumorspheres also had increased levels of Gln; deprivation of the amino acid resulted in inhibition of sphere-forming properties, reduction in the ALDH^+^CSCs, diminished tumor-initiating capacity in vivo, and increased radiosensitization. In contrast, Gln depletion in the LNCaP cells did not have such severe effects. The metabolic reprogramming towards Gln has been associated with high *MYC* expression levels [43], a finding that was also confirmed in this study [42]. 

Another interesting study investigated the role of Gln metabolism on the stem-like side populations (SPs) of the A549 NSCLC and AsPC-1 pancreatic cancer cell lines [44]. Gln deprivation decreased the stem-like SP in both cell lines, supporting the importance of this nutrient in the maintenance of stemness properties. Moreover, blocking of Gln metabolism by the drug L-asparaginase, which enzymatically depletes Gln, also led to the decrease in stem-like SP in A549 cells [44]. Both Gln deprivation and depletion resulted in the downregulation of the stemness markers *SOX-2* and *ABCG2*, both on the transcriptional and translational level. Gln replenishment reversed the effect of its deprivation in the stem-like SP population in the A549 cells, which recovered, while it also upregulated the expression of *SOX-2* and *ABCG2* [44]. Further investigation of the mechanisms of action of Gln deprivation revealed an association with GSH synthesis and ROS balance that affected the stemness properties of CSCs. Specifically, in the absence of Gln, A549 cells showed GSH reduction, attenuation of the antioxidant system, and an increase in ROS [44]. The above results that highlighted the importance of Gln in CSCs were also confirmed by in vivo experiments. A549 cells cultured under Gln deprivation conditions were inoculated in mice and tumors developed only in one mouse out of twelve, an observation that proposed Gln deprivation as a strategy that severely impairs in vivo tumorigenicity [44]. 

The association of Gln metabolism with the expression of stemness properties and the evasion of chemotherapy-induced senescence has also been examined in breast cancer [45]. Specifically, MCF-7 cells treated with doxorubicin underwent senescence, a cellular response that is characterized as therapy-induced senescence (TIS) [46]. When TIS cells were kept under the above culture condition for prolonged periods, they formed senescence-resistant colonies, showing an increased CD44^+^/CD24^−/low^ subpopulation compared to parental MCF-7 cells [45]. Gln deprivation resulted in the reduction of cells that could escape TIS, a phenomenon that was attributed to CSCs. Further analysis revealed that reduction of Gln concentration led to the decrease in CD44^+^/CD24^−/low^ cells, while supplementation with Gln was accompanied by a significant increase in these cells [45]. Furthermore, TIS cells overexpressed the Gln transporter *SLC1A5* supporting high Gln metabolism compared to parental MCF-7 cells, as well as the stem cell marker *NANOG*. Pharmacological inhibition of SLC1A5 reduced the CD44^+^/CD24^−/low^ subpopulation, indicating a Gln dependency for their survival, while it also promoted their escape from TIS [45].

Finally, a new study used magnetic resonance imaging (MRI) to assess Gln uptake in mouse xenografts of HT29 colorectal cancer cells [47]. Higher Gln uptake was associated with higher expression of the Gln transporters ASCT2 and SLC38A2, glutaminase, and the CSC markers CD44 and CD166. On the contrary, regions with lower Gln uptake exhibited lower expression of these transporters and CSC markers. Pharmacological inhibition of the ASCT2 also reduced Gln uptake, as measured by MRI [47]. The same group had previously reported that Gln metabolism was involved in the differential effects of metformin in CSCs isolated from different colorectal cancer cell lines [48]. Metformin treatment resulted in significant reduction in the CD133^+^/CD44^+^ CSCs of certain cell lines (deemed metformin sensitive), but not in others (deemed metformin resistant). Further experiments showed that the metformin-induced AMPK (adenosine monophosphate-activated protein kinase)-dependent mTOR (mammalian target of rapamycin) pathway was involved in the regulation of the metformin-sensitive HT29 CSCs. The metformin-sensitive HT29 cells were also more dependent on OXPHOS than the metformin-resistant SW620 cells. The CSC-suppressing effect of metformin was induced in SW620 cells and enhanced in HT29 cells under Gln deprivation conditions, where tumorspheres from either cell line could not survive. The expression of the transporter ASCT2 was higher in the SW620 compared to HT29 cells, an observation that suggested a higher ability of the former to utilize Gln. Knock-down of *ASCT2* in the SW620 cells significantly decreased the CD133^+^/CD44^+^ CSCs upon treatment with metformin, proposing that inhibition of the Gln pathway could be an effective complementary treatment to metformin to enhance its CSC-suppressing effect, especially in resistant cells [48]. 

Gln metabolism has emerged as an important metabolic pathway in the regulation of CSCs, suggesting that its inhibition or Gln deprivation could be an attractive therapeutic choice in cancer treatment. 

### 3.3. Lipid Metabolism

Lipids encompass a large heterogeneous family of organic compounds that are essential for a multitude of cellular functions, including energy production, membrane biosynthesis, and signal transduction. Lipid metabolism is dysregulated in cancer sustaining tumor growth, progression, and metastasis [49]. Increasing evidence reveals that lipid metabolism is also associated with the stemness properties of CSCs, which rely heavily on de novo lipogenesis and lipid oxidation, as indicated by the upregulation of key enzymes of these processes [36]. 

Indeed, the stearoyl-CoA desaturase 1 (SCD1) enzyme that regulates the conversion of saturated into monounsaturated fatty acids (MUFAs) was associated with stemness in ovarian, breast, and liver cancer, as its overexpression promoted CSC proliferation while preventing apoptosis [50]. The sterol regulatory element binding protein 1 (SREBP1) regulates fatty acid and cholesterol biosynthesis. When it was overexpressed, it could maintain the stemness properties of CD44^+^CD24^−^ESA^+^ breast CSCs isolated from the MCF10AT cell line and clinical specimens, and it could promote tumor progression [51].

Colon CSCs isolated from the HCT-166 cell line contained more unsaturated lipids and fatty acids than their non-CSC counterparts, and this lipid abundance was essential for maintaining their stemness properties [52]. Inhibition of SCD1 resulted in a decrease in the levels of unsaturated lipids and impaired the capability of CSCs to form spheres [52]. 

Moreover, MUFAs can affect CSC generation and their stemness properties [53]. It has been reported that CSCs from glioblastoma and ovarian tumors showed higher levels of MUFAs than non-CSCs [54,55]. Pharmacological inhibition of the pathways associated with MUFA biosynthesis in tumorspheres generated from the U87 glioblastoma cell line [54], as well as inhibition of SCD1 in tumorspheres from the ovarian COV362 and OVCAR5 cells [55], resulted in the reduction of CSC stemness properties and survival, suggesting that lipid desaturation could be a CSC biomarker. 

Similarly, the enzyme fatty acid synthase (FASN) that mediates fatty acid synthesis has been reported to be highly active in the GSC lines G144 and G179, as well as in tumorspheres generated from glioma tissue samples surgically resected from patients after their diagnosis [56]. Increased FASN expression maintained the stemness and invasiveness properties of GSCs. Inhibition of the enzyme’s activity resulted in the suppression of de novo lipogenesis and, subsequently, in the inhibition of cell proliferation, downregulation of stemness markers, and impaired migratory ability of GSCs [56]. 

Overall, the alterations in the lipid metabolism of CSCs play an essential role in their survival and maintenance through the modulation of key signaling pathways, as it has been reviewed elsewhere [57]. Targeting these alterations could achieve CSC elimination and improve the outcome of anticancer therapies.

## 4. Autophagy/Mitophagy and CSC Metabolism

Autophagy is a highly conserved cellular process that involves the breakdown of intracellular components, including molecules and organelles, via lysosome-mediated degradation [58]. The autophagy products are used to support cellular homeostasis, development, and survival. Disruption of the autophagic process can contribute to tumor development and growth, and clinical trials are currently underway to investigate its role in cancer therapy [58]. A link between autophagy and cancer metabolism has also been reported, serving as a mechanism for the metabolic adaptation of tumor cells to nutrient starvation [59]. Autophagy has been associated with stemness in many tumor types, enabling CSCs to survive in hypoxic, poor in nutrients niches [60]. Several comprehensive reviews describe current knowledge on autophagy and CSCs [60,61,62]; here, we briefly discuss some studies that reveal the crosstalk between autophagy and CSC metabolism.

The expression of CD133, a well-known stem cell marker, was found to regulate autophagy in GSCs in a glucose-deprived environment [63]. GSCs expressing CD133 were isolated from the F98-CD133 and C6-CD133 cell lines. CD133^+^ cells exhibited enhanced survival and reduced apoptosis compared to CD133^−^ cells under glucose deprivation through the activation of autophagy-associated genes. Further investigation revealed that CD133 was more abundant in the cytoplasm in starvation conditions, whereas it was membrane-bound under normal glucose levels [63]. This observation suggested that, during starvation, CD133 was released from the membrane to the cytoplasm, participating in the formation of autophagosomal membrane fusion and promoting autophagy to compensate for nutrient deprivation. This was not an option for CD133^−^ cells. The above study proposed that targeting CD133-signaling and autophagy in glioma could improve anti-cancer treatment. 

Autophagy has also been linked to the metabolic mechanisms of the SCs in a study by Viale et al., in PDAC [32]. Transcriptomic and metabolomic analyses of these stem-like cells revealed an increased reliance on autophagy for their survival [32]. The autophagic marker microtubule-associated protein light-chain 3 was highly expressed in SCs, wherein they also exhibited increased autophagosome formation compared to KRAS-expressing tumorspheres [32]. Autophagy inhibition increased the metabolic stress in SCs by affecting mitochondrial activity, while it decreased their spherogenic potential and survival [32]. The above study concluded that mitochondria electron transport activity was strongly dependent on autophagic processes.

Mitophagy, the process by which aged and damaged mitochondria undergo autophagy, has been associated with CSC metabolic reprogramming and survival, especially under stressful conditions, such as hypoxia and chemotherapy [60]. It is regulated by several signaling pathways, including the B-cell lymphoma 2/adenovirus E1B interacting 19 kDa-interacting protein 3 (BNIP3) and BNIP3-like (BNIP3L) pathways.

An interesting study showed that interferon-stimulated gene 15 (*ISG15*), an ubiquitination-like modifier, and the post-translational modification it regulates, known as ISGylation, were upregulated in PaCSCs [64]. RNA-sequencing revealed an association between the expression of *ISG15*, stemness genes, and genes associated with mitochondrial processes, including OXPHOS [64]. Genetic ablation of *ISG15* in the PaCSCs using CRISPR led to accumulation of dysfunctional mitochondria, reduced OXPHOS and impaired glycolysis. Further experiments confirmed that loss of *ISG15* led to an impairment of mitophagy and an increase in autophagosomes and autophagy flux, possibly as a compensatory mechanism [64]. The same research group had shown before that PaCSCs showed metabolic plasticity in the presence of metformin, which allowed drug-resistant cells to outgrow, as it was described in [34]. However, PaCSCs with *ISG15* loss were highly sensitive to the mitochondrial inhibitor in vitro and in vivo, indicating a diminished metabolic plasticity [64].

Expression of the hepatitis B virus x protein (HBx) is a predisposing factor for HCC and promotes cancer stemness. In a recent study, the authors confirmed that expression of HBx induced a cancer stemness phenotype and promoted a metabolic shift towards glycolysis in HCC in vitro and in vivo [65]. By inhibiting glycolysis in HBx-expressing cells, they further showed that this metabolic process was important in maintaining cancer stemness induced by HBx in HCC. Subsequent experiments demonstrated that CSCs had a high level of BNIP3L-dependent mitophagy, and the authors were able to link this to HBx expression. They concluded that HBx induced BNIP3L-dependent mitophagy, which, in turn, metabolically reprogrammed HCC cells towards glycolysis, supporting an enhanced cancer stemness phenotype [65].

The above studies support the idea that autophagy/mitophagy can promote CSC survival and stemness through metabolic reprogramming and suggest that blocking them may be a new therapeutic intervention against this highly tumorigenic population. 

## 5. Stemness Pathways Regulate Metabolic Reprogramming and Adaptation in CSCs

**Figure 4:**
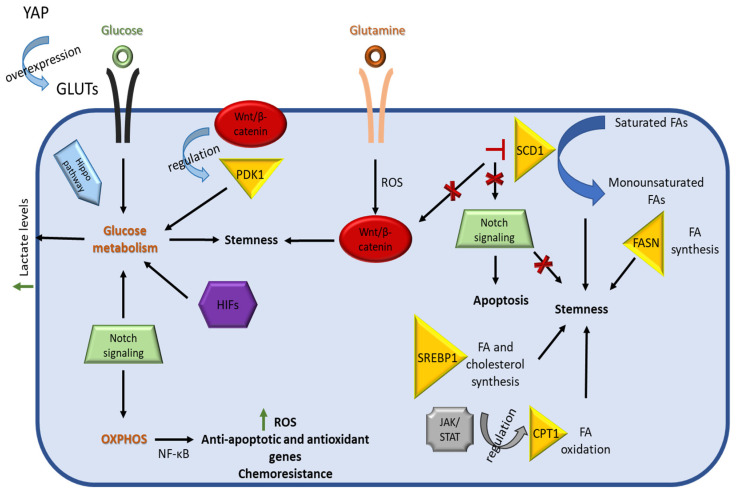
Schematic representation of pathways associated with CSC metabolic reprograming. The Hippo pathway regulates CSC metabolism as YAP upregulates GLUTs expression and subsequently glucose uptake, contributing to stemness. The Wnt/β-catenin pathway promotes stemness by directing cancer stem cells towards glucose metabolism via the expression of PDK1. This pathway is also regulated by a ROS-dependent mechanism that is associated with Gln metabolism. Notch signaling directs CSCs towards glycolysis or OXPHOS, depending on their niche. The Notch-mediated activation of OXPHOS through NF-κΒ leads to an increase in ROS levels, expression of anti-apoptotic genes, and chemoresistance in CSCs. Lipid metabolism in CSCs regulates their stemness properties, with the enzyme SCD1 playing a major role through the conversion of saturated FAs to monounsaturated FAs. Pharmacological inhibition of SCD1 results in the inhibition of both Wnt/β-catenin and Notch signaling pathways. The JAK/STAT pathway is linked with fatty acid oxidation (FAO) through the regulation of the expression of the rate limiting enzyme CPT1, among others.

As it was extensively reported in the sections above, the metabolic networks of CSCs are interlinked with their stemness properties. Several well known signaling pathways that support self-renewal and survival in CSCs, including Hippo, WNT/β-catenin, JAK/STAT, and Notch, seem to be also involved in the regulation of the metabolic reprogramming of these cells (Figure 4).

### 5.1. Hippo Pathway

Hippo signaling is an evolutionarily conserved pathway and a master regulator of cell proliferation and cell fate during organ development [66]. The major mediators of this pathway are the mammalian STE20-like protein kinase-1 and -2 (MST1 and MST2), which phosphorylate and activate the large tumor suppressor kinases-1 and -2 (LATS1 and LATS2), which, in turn, inhibit the activity of the transcriptional activators yes-associated protein (YAP) and transcriptional co-activator with PDZ-binding motif (TAZ). The inactivated YAP/TAZ either remains in the cytoplasm or it gets marked for degradation by ubiquitination. When the Hippo pathway is inactive, the YAP/TAZ remains unphosphorylated and translocates to the nucleus, where it regulates gene expression after association with the DNA-binding protein TEAD [66]. In cancer, the Hippo signaling is dysregulated, promoting tumorigenesis, cell invasion, metastasis, and resistance to therapies [67]. Recent studies have also revealed an important role for the Hippo network in CSC biology, including its association with stemness, the epithelial to mesenchymal transition (EMT), and drug resistance [68]. 

It has also been demonstrated that YAP/TAZ activation regulates metabolism and metabolic reprogramming of CSCs [69]. In the metastatic colorectal cancer cells 116-LM, YAP activation led to higher glucose uptake and increased aerobic glycolysis compared to their non-metastatic counterparts (HCT166 cells) through the upregulation of *GLUT3* and other glycolytic enzymes [70]. *GLUT3* overexpression in the HCT116 cells resulted in higher expression of stemness-related genes and increased tumorsphere formation, while *GLUT3* silencing in the 116-LM cells suppressed their metastatic properties and reduced the expression of stemness-associated transcription factors [70]. The *GLUT3*-induced invasiveness and stemness properties were attributed to a YAP-depended mechanism, as silencing of YAP signaling suppressed these properties [70]. 

In breast cancer, YAP/TAZ activity has been linked to high-grade tumors and high CSC content, reflecting its correlation with aggressiveness [71]. Bioinformatic analysis of clinical data from more than 3600 primary mammary tumors revealed an association between the expression of genes linked with high glucose metabolism, higher tumor grade, and expression of stemness genes and YAP/TAZ activity [72]. This analysis supported the idea that, during tumor progression, the elevated activity of YAP/TAZ leads to metabolic reprogramming towards aerobic glycolysis [72]. In a different study, it was shown that CD44^+^CD24^−/low^ breast CSCs isolated from several cell lines had a high expression of a long non-coding RNA, lncROPM, which upregulated phospholipid metabolism and free fatty acid production, leading to activation of the Hippo pathway and maintenance of the stemness properties [73]. More specifically, lnROPM regulated the expression of phospholipase A_2_ (PLA_2_G16), leading to the production of free fatty acids and especially arachidonic acid. Knockdown of lncROPM in CSCs significantly decreased the expression of stemness-related genes and mammosphere size, while its overexpression in non-CSCs promoted the expression of such genes and increased the MFE. Lipidomic analysis of the lncROPM-knocked-down CSCs and the lncROPM-overexpressing non-CSCs revealed that arachidonic acid was the most significantly altered metabolite between the two groups. Arachidonic acid administration resulted in the expression of stemness-related genes in the knocked-down breast CSCs through the activation of both the Hippo/YAP and the Wnt/ β-catenin signaling [73].

The above described studies provide substantial evidence to support the regulation of CSC metabolism by the Hippo pathway, yet the underlying mechanisms are still unclear and need further investigation [68].

### 5.2. Wnt/β-Catenin Pathway

The Wnt/β-catenin signaling cascade is also evolutionary conserved, as it is critical for numerous physiological processes including cell fate, proliferation, migration and polarity in development, and tissue homeostasis [74]. Dysregulation of this pathway is a hallmark of many cancers, where it has been linked with tumorigenesis, tumor metastasis, and immunoevasion [74]. 

The Wnt/β-catenin pathway is also involved in the reprogramming of cancer metabolism, where it directs cells into glycolysis and away from mitochondrial OXPHOS, through the regulation of *PDK1* expression and by reducing the conversion of pyruvate into acetyl-CoA [75]. Blocking of the Wnt/β-catenin pathway in colon cancer cells decreased their dependence on aerobic glycolysis by downregulating the key glycolytic enzyme PDK1 [76]. Aberrant Wnt activity has been associated with cancer cells endowed with stem cell properties, and it is one of the primary targets to eradicate these cells [77].

Furthermore, the Wnt pathway seems to be a mediator of the effects of metabolic changes on CSC survival, as described in the following studies. MDA-MB-231 and MCF-7 mammospheres and PC3 and LNCaP prostate tumorspheres were grown in medium with no, low, or high glucose and were treated with the Wnt/β-catenin pathway inhibitor sFRP4 [78]. The tumorspheres grown with no or low glucose were more susceptible to the inhibitor’s activity that resulted in sphere disruption, confirming the important role of this nutrient in CSC survival. Additionally, the administration of sFRP4 resulted in the decrease in glucose and glutamine uptake by the cells, while it promoted apoptosis. Thus, targeting the Wnt/β-catenin pathway can reduce CSC viability through modulation of glucose metabolism [78].

The crosstalk of Wnt signaling with lipid metabolism was investigated in colon CSCs [79]. Pharmacological inhibition of the lipid desaturation enzyme SCD1, which leads to altered lipid metabolism, resulted in decreased number of CSCs and abolishment of sphere formation generated by three colon cancer cell lines (HT29, HCT15 and SW480), while it did not affect non-CSCs. SCD1 inhibition was also associated with the suppression of Wnt genes, suggesting that targeting lipid metabolism in colon CSCs may lead to their elimination through downregulation of Wnt signaling [79]. 

The Wnt/β-catenin pathway has also been linked with the stemness properties of CSCs through glutamine metabolism. Specifically, in the stem-like side populations isolated from the A529 NSCLC and the AsPC-1 pancreatic cancer cell lines, as well as in the GSC11 and GSC23 GSCs, glutamine activated the Wnt/β-catenin pathway through a ROS-mediated mechanism and upregulated the expression of stemness genes. Glutamine deprivation or inhibition of glutamine metabolism led to an increase in ROS levels and inactivation of β-catenin, decreasing stemness properties [44]. 

The above studies suggest that the Wnt/β-catenin pathway can be modulated in CSCs by nutrient metabolism to affect the cells’ viability and stemness. 

### 5.3. JAK/STAT Signaling

Janus kinases (JAKs) are intracellular tyrosine kinases that mediate the phosphorylation of STAT proteins, leading to the translocation of STATs into the nucleus and the activation of gene expression. The JAK/STAT pathway is critical for a multitude of physiological processes involved in development and tissue homeostasis, such as hematopoiesis, stem cell maintenance, immunity, tissue repair, and inflammation [80]. An increasing number of studies suggest that aberrant regulation of the JAK/STAT pathway is associated with various cancers [80]. The role of JAK/STAT signaling in cancer cell metabolism has been reported and, particularly, in the functions of STAT3 and STAT5 in the regulation of metabolism-related genes [81], while data in CSCs is limited.

A recent study shed light onto the association between JAK/STAT3 signaling and breast CSC metabolism [82]. Treatment of the breast cancer cell line HCC1937 with the pan-JAK inhibitor AZD1480 resulted in the decrease in viability of CSCs, in contrast to non-CSCs [82]. Similar results were obtained when the same inhibitor was administered to MCF-7 tumorspheres, leading to suppression of sphere formation. RNA-sequencing analysis of treated tumorspheres revealed the downregulation of lipid metabolic genes, especially genes associated with fatty acid oxidation (FAO), with one of them being carnitinepalmitoyl transferase 1 (CPT1), the rate limiting enzyme for FAO [82]. Metabolomic analysis of the CSC-enriched Hs578T and MDA-MB-436 breast cancer cell lines showed higher levels of FAO metabolites compared to CSC-poor cell lines [82]. *STAT3* knock-down reduced the expression of FAO genes, including *CPT1B,* in MDA-MB-468 tumorspheres and inhibited their self-renewal [82]. Interestingly, clinical data from breast cancer patients revealed higher expression of *CPT1B* in breast carcinomas compared to healthy tissues, while it was also found elevated in recurrent tumors. High CPT1B levels also correlated with poor patient outcome and were negatively associated with therapeutic response [82]. Based on these data, the authors concluded that the STAT3-CPT1B-FAO axis is critical for breast cancer cell stemness and therapy resistance.

### 5.4. Notch Pathway

The Notch signaling pathway is a highly conserved pathway that orchestrates cell fate decisions during development. It consists of the Notch cell-surface receptors that bind transmembrane ligands expressed on neighboring cells to mediate cell–cell communication. Binding of the Notch ligands results in the proteolytic cleavage of the intracellular domain of the receptor, its translocation to the nucleus, binding to the CLS protein (CBF1, Suppressor of Hairless, Lag-1), and, finally, to the transcriptional activation of targeted genes [83]. Notch signaling also plays a major role in cancer, as mutations in genes involved in this pathway have been identified in various cancer types, where they function as oncogenes or tumor suppressors depending on cell context [84]. Several studies have confirmed that the Notch pathway is crucial in governing self-renewal and maintenance in CSCs [85]. Emerging evidence suggests that it may also be a mediator of CSC metabolism. 

In glioblastoma, metabolic adaptations that supported the survival and growth of GSCs in diverse niches were associated with heterogeneous activation of Notch signaling [86]. The researchers identified distinct GSC populations in patient-derived cultures that were marked by high expression of the stemness marker CD133 or highly activated Notch status. The CD133^hi^ GSCs were located in hypoxic niches and mainly relied on anaerobic glycolysis, while the Notch^hi^ cells resided in perivascular niches using mostly OXPHOS for their energy needs [86]. Ectopic activation of the Notch pathway in the CD133^hi^ cells led to their metabolic reprogramming through suppression of anaerobic glycolysis, which rendered the cells vulnerable to hypoxia [86].

A previously described study linking SCD1 inhibition with the suppression of the Wnt pathway showed that Notch signaling was affected, too [79]. Pharmacological inhibition of SCD1 selectively induced apoptosis in colon CSCs without affecting non-CSCs. It was assumed that this occurred via the downregulation of the Notch pathway, since related genes were found suppressed. These data led to the proposition that SCD1 could be a specific target in colon CSCs, and its inhibition could improve the clinical outcome of conventional therapies [79]. 

Notch signaling also regulates CSC survival in triple negative breast cancer (TNBC) through activation of mitochondrial metabolism [87]. Oncogenic activities were attributed to Notch signaling through the induction of OXPHOS and the activation of the NF-κΒ pathway that led to the transcription of anti-apoptotic genes. Inhibition of Notch signaling in MDA-MB-231-derived mammospheres decreased OXPHOS, and it was suggested that this could be an effective way to target CSC metabolism and reduce their survival [87]. 

The aforementioned studies reveal a new role for the Notch pathway in the regulation of CSCs through its intersection with CSC metabolism. Further work should elucidate the downstream effectors of Notch signaling on the metabolic network of these cells.

## 6. Hypoxia and CSC Metabolism

Low oxygen level (hypoxia) is a major stressor to cells, which have developed adaptive mechanisms to manage it. Hypoxia-inducible factors (HIFs) are the key mediators of cellular adaptation to this condition and regulate the expression of genes involved in cell proliferation, apoptosis, metabolism, and invasion [88]. HIF is a heterodimeric transcription factor consisting of an oxygen sensitive a subunit (HIF-1α, -2α or -3α) and a constitutively expressed β subunit (HΙF-1β), with HIF-1α being the main regulator of glycolytic transporters and enzymes in response to hypoxia [89]. It is well established that the hypoxic tumor microenvironment has a prominent role in tumor progression and resistance to therapy [90]. Notably, CSCs prefer to reside in hypoxic niches, where the adaptive mechanisms induced in the cells, including metabolic alterations, play an important role in sustaining their stemness potential [91]. Under such conditions, a switch from OXPHOS to glycolysis is mandatory for the CSCs to fulfill their energy demands. Several recent studies have shed light into the interplay between hypoxia and metabolism to promote cancer stemness.

The roles of ubiquitin-specific protease 22 (USP22) and HIF-1α were investigated in promoting stemness and metabolic alterations in HCC in a recent study [92]. Overexpression of USP22 in HCC cell lines, under hypoxic conditions, significantly enhanced glycolysis, as it upregulated the mRNA expression of key glycolytic enzymes (*HK2*, *PDK1,* and *ENO1*). It also promoted stemness properties in these cells, which was manifested by an increase in tumorsphere formation and in drug resistance, as well as an enhanced migratory ability [92]. Knock-down of USP22 resulted in the downregulation of genes associated with both glycolysis and stemness and suppression of stemness properties. The use of the glycolysis inhibitor 2-DG abolished the effect of USP22 in stemness under hypoxic conditions. Further experiments revealed that USP22 actions were mediated through the deubiquitination and subsequent stabilization of HIF-1α and its transcriptional activity. HIF-1α knock-down resulted in the abrogation of the USP22-enhanced cancer stemness and glycolysis under hypoxic conditions [92]. The authors proposed that USP22 may be a potential target in HCC and presented data from in vivo experiments to validate their hypothesis. Overall, this study highlighted the associations between hypoxia, cancer stemness, and metabolism and also provided a mechanism by which HIF1α/USP22 promote glycolysis and stemness in HCC [92]. 

The association between HIF-1α, stemness, and metabolism was also studied in breast cancer [93]. Knock-down of the CSC marker CD44 in a number of breast cancer cell lines (MDA-MB-231, Hs578T, MCF7 and 293T) led to a decrease in the mRNA expression of several glycolytic genes, including *GLUT-1* and *LDHA*. Additionally, the glucose uptake and lactate production were decreased, while the endogenous cellular oxygen consumption was increased, confirming a switch towards OXPHOS in these cells [93]. The effect of CD44 was linked to HIF-1α expression and its transcriptional activity upon the LDHA promoter. Specifically, CD44 ablation led to downregulation of HIF-1α and decreased binding of the transcription factor to the LDHA promoter. Interestingly, CD44 silencing had the opposite effects on LDHB, which was upregulated [93]. The authors concluded that the breast CSC marker CD44 was important in the regulation of cancer cell metabolism by modulation of the LDH isoenzymes levels through the HIF-1α [93]. 

In a different study, the extended use of dimethyl-2-ketoglutarate (DKG), a cell membrane-permeable α-KG analogue that stabilizes HIF-1α, could reprogram breast cancer cells to acquire stem-like characteristics [94]. Specifically, DKG treatment resulted in higher tumorsphere formation, enrichment of the CD44^high^/CD24^low^ subpopulation, and higher expression of pluripotency genes, including *OCT4* and *NANOG,* in MDA-MB-231, MCF7, T47-D, and MDA-MB-468 breast cancer cells. It also enhanced the tumorigenic properties of the MDA-MB-231 cells in vivo. Knock-down of HIF-1α significantly reduced the DKG-dependent induction of OCT4 and the CD44^high^/CD24^low^ subpopulation [94]. Further investigation of the metabolic pathways that were affected by the DKG treatment revealed that the treated cells had HIF-1α-dependent increased expression of genes associated with glucose metabolism (*GLUT1* and *PDK1*), while the expression of genes associated with the mitochondrial electron transport chain was reduced [94]. Thus, it was proposed that metabolic rewiring by elevated levels of DKG led to stabilization of HIF-1α and reprogramming of breast cancer cells into a stem-like state [94].

These representative studies suggest that hypoxia and metabolic reprogramming can sustain CSCs and, thus, targeting the related factors (e.g., HIFs) and/or pathways should be taken under consideration when devising therapeutic strategies against cancer.

## 7. CSC Metabolism and Drug Resistance

### 7.1. Drug Resistance in CSCs

Increased drug resistance is a key feature of CSCs, and it has been attributed to several adaptive mechanisms that these cells have developed to survive the stress from drug exposure. These mechanisms include the upregulation of DNA repair mechanisms with overactivated cell-cycle checkpoints and overexpression of DNA damage repair proteins, as well as the increased expression of transmembrane drug-efflux pumps and the adoption of a quiescent state, where CSCs reversibly arrest in the G0 phase and exhibit basal metabolic activity [95]. It has been proposed that the metabolic plasticity of quiescent CSCs is associated with their increased chemoresistance [96]. 

### 7.2. Metabolic Reprogramming of CSCs towards OXPHOS Is Associated with Drug Resistance

Since the metabolic rewiring of CSCs is closely interlinked with their enhanced drug resistance, deciphering their metabolic fingerprint to uncover potential targets has attracted much attention as a means for CSC elimination and coping with tumor recurrence. Interestingly, it has been reported by several groups that OXPHOS is the main regulator of drug resistance in CSCs from different types of cancer [97,98,99,100,101,102,103]. 

The switch of CSC metabolism towards OXPHOS results in high levels of mitochondrial ROS and resistance to oxidative stress through anti-oxidant mechanisms [104]. In an in vitro system enriched in ovarian CSCs, ROS levels were upregulated, compared to the PA1 parental cells, and induced the expression of PGC-1α, a master regulator of metabolism and energy homeostasis [97]. Tumorsphere formation promoted the upregulation of OXPHOS-related genes, an increase in mitochondrial mass, and a decrease in mitochondrial activity presumably via induced mitochondrial fission [97]. PGC-1α was shown to mediate resistance to cisplatin and paclitaxel, while ROS scavenging in spheres led to their sensitization to drug treatment. Finally, the researchers showed that ROS-induced PGC1α mediated the chemoresistance of ascites-derived cancer cells enriched in ALDH^+^ CSCs [97].

Similar results regarding the association of high mitochondrial mass with a stemness phenotype were obtained in breast cancer [98]. High mitochondrial mass was correlated with the expression of stemness markers in subpopulations of MCF-7, MDA-MB-231 cells, and primary metastatic breast cancer samples. These mitochondria-rich subpopulations also exhibited increased MFE in vitro and tumor initiating capacity in vivo, as well as enhanced resistance to paclitaxel treatment [98].

MYC and the myeloid cell leukemia-1 protein (MCL1), an anti-apoptotic protein, were found to be overexpressed in drug-resistant TNBC patients after therapy, in paclitaxel-resistant MDA-MB-436 and SUM159PT cells and in CSC-enriched mammospheres generated from the parental cell lines [99]. In a series of experiments, the authors showed that MYC and MCL1 co-operatively enhanced OXPHOS and ROS generation, which further increased the CSC-subpopulation in mammospheres derived from the paclitaxel-resistant cells, indicating the association of these factors with chemoresistance [99]. The enhancement of CSCs by MYC and MCL1 was mediated through hypoxia and HIF-1α overexpression, as knock-down of HIF-1α abrogated the induction of CSCs, proposing this factor as a target for drug sensitization in chemotherapy-resistant TNBC patients [99]. 

Sirtuin 1 (SIRT1) is a NAD-dependent histone deacetylase that has a central role in regulation of gene expression, stemness maintenance, and metabolism [105]. It is also important in the development of cancer resistance to tyrosine kinase inhibitors (TKI) [106]. *SIRT1* was found to be overexpressed in the gefitinib (an EGFR-TKI) resistant PC9 and HCC827 lung cancer cells lines, which were also significantly enriched in CSCs compared to the parental cells [100]. The CSC fractions of the resistant cell lines exhibited higher OXPHOS than their parental counterparts and relied on this process for their survival. Combined administration of the TKI and OXPHOS inhibitors rendered the cells more susceptible to the drug. Knock-down or inhibition of *SIRT1* in the resistant cell lines reduced OXPHOS, the tumorsphere formation capacity, and the ALDH1^+^ CSC fraction and enhanced drug-sensitivity [100]. These results suggested that *SIRT1* promoted OXPHOS and subsequent CSC enrichment in TKI-resistant lung cancer. Clinical data from lung cancer patients indicated that higher expression of *SIRT1* and OXPHOS-associated proteins correlated with tumor recurrence and poor survival, supporting that OXPHOS inhibitors could be a part of a combination therapy for better clinical outcome in these patients [100].

LSC-enriched populations, resistant to chemotherapy, were isolated from primary AML samples and were found to be metabolically quiescent with lower levels of ROS compared to non-SCs [101]. Metabolic analysis showed that the LSCs were dependent more on OXPHOS, rather than glycolysis, for the generation of ATP [28,101]. Gene expression analysis of these cells revealed the upregulation of *BCL-2* (B-cell lymphoma-2), a gene commonly found overexpressed in cancers [103]. Pharmacological inhibition of *BCL-2* resulted in OXPHOS impairment and selective eradication of chemoresistant LCSs, without having any toxicity effect to normal cells from healthy donors [101].

Stem-like gastric cancer cells, generated through in vitro chronic metabolic stress, reprogrammed their metabolism towards increased OXPHOS compared to parental cells [107]. Surprisingly, these CSCs had reduced ROS levels, which were associated with their resistance to 5-FU. Low levels of ROS in these CSCs were maintained through the transcription factor *FoxM1* that controlled the ROS detoxification gene *PRX3,* as well as through an increased fatty acid oxidation-mediated NADPH regeneration. Both *FoxM1* and the enhanced NADPH regeneration were shown to mediate drug resistance in CSCs [107]. Additionally, upregulation of *FoxM1* was associated with the prediction of poor survival in patients with different types of cancer. It was proposed that a mitochondrial ROS homeostasis-targeted approach in CSCs could constitute a therapeutic strategy against these therapy-resistant cells [107].

In pancreatic tumorspheres from PDAC cell lines (PC-1, BxPC-3, HPAF-II) enriched in CSCs, the maintenance of a quiescent metabolic state with a reduced glycolytic activity was associated with increased chemoresistance [102]. However, the authors also noted that this slow metabolic potential could also be regarded as a metabolic vulnerability, which would prevent CSCs to respond and adapt to extremely unfavorable stressors [102].

Finally, in chemoresistant HEP-G2 (rHEP-G2) cells with a CSC phenotype, metabolic reprogramming from glucose to glutamine dependency via mitochondria led to the adoption of a quiescent state from these cells [108]. The use of metformin, a mitochondrial-specific antagonist, led to re-sensitization of the rHEP-G2 cells to doxorubicin, offering a new therapy approach via targeting CSC metabolism [108].

The inability of common drugs to fully eradicate CSCs leads to tumor recurrence and poor patient survival, rendering efficient targeting of CSCs a matter of high importance. Inhibiting OXPHOS has gained a great interest as a means to overcome CSC drug resistance. A number of pharmacological agents that target OXPHOS are under investigation in ongoing clinical trials. Several FDA-approved agents, such as salinomycin, erythromycins, tetracyclines, and glycylcyclines, selectively eradicate CSCs through OXPHOS inhibition [109,110]. Notably, the inhibition of mitochondrial respiration can eliminate not only CSCs that exhibit increased OXPHOS, but also glycolytic CSCs in different types of cancer [34,111,112]. 

## 8. Conclusions

Drug resistance is still a major challenge for the treatment of cancer, and its association with cancer cells with stem-like properties is now well established. CSCs have developed a wide repertoire of mechanisms to evade chemotherapy; one of them is metabolic plasticity that allows them to switch between glycolysis and OXPHOS, depending on stimuli from their environment. Several signaling pathways, such as Hippo, Wnt/β-catenin, Notch, and JAK/STAT, are interlinked with the metabolic flexibility of CSCs, underscoring the complex regulation of this trait. However, more studies are needed for clarifying the role of metabolic plasticity of CSC in cancer progression, metastasis, chemoresistance, and tumor recurrence.

Metabolic targeting of CSCs remains a challenging goal, as, in most cases, inhibition of one metabolic pathway leads the cells to enhance other metabolic processes for their survival. As CSCs cover their energy needs mainly through OXPHOS and glycolysis, simultaneous targeting of these two pathways could be an alternative and more effective therapeutic approach for their complete eradication.
